# Barriers experienced by families new to Alberta, Canada when accessing routine-childhood vaccinations

**DOI:** 10.1186/s12889-023-16258-7

**Published:** 2023-07-12

**Authors:** Madison M. Fullerton, Margaret Pateman, Hinna Hasan, Emily J. Doucette, Stephen Cantarutti, Amanda Koyama, Amanda M. Weightman, Theresa Tang, Annalee Coakley, Gillian R. Currie, Gabriel Fabreau, Cora Constantinescu, Deborah A. Marshall, Jia Hu

**Affiliations:** 1 Faculty of Nursing, University of Calgary, Calgary, AB Canada; 2 19 to Zero Inc, Calgary, AB, Canada; 3 Department of Pediatrics, Cumming School of Medicine, University of Calgary, Calgary, AB Canada; 4 Department of Community Health Sciences, Cumming School of Medicine, University of Calgary, Calgary, AB Canada; 5 Alberta Children’s Hospital Research Institute (ACHRI), University of Calgary, Calgary, AB Canada; 6 Calgary Catholic Immigration Society, Calgary, AB Canada; 7 Habitus Consulting Collective Inc, Calgary, AB Canada; 8 Mosaic Refugee Health Clinic, Calgary, AB Canada; 9 Department of Family Medicine, University of Calgary, Calgary, AB Canada; 10 Department of Medicine, Cumming School of Medicine, University of Calgary, Calgary, AB Canada; 11 Pediatric Infectious Diseases, Department of Pediatrics, Cumming School of Medicine, University of Calgary, Calgary, AB Canada

**Keywords:** Routine childhood vaccination, Newcomer, Immunization, Health equity, Access

## Abstract

**Background:**

As Canada and other high-income countries continue to welcome newcomers, we aimed to 1) understand newcomer parents’ attitudes towards routine-childhood vaccinations (RCVs), and 2) identify barriers newcomer parents face when accessing RCVs in Alberta, Canada.

**Methods:**

Between July 6th—August 31st, 2022, we recruited participants from Alberta, Canada to participate in moderated focus group discussions. Inclusion criteria included parents who had lived in Canada for < 5 years with children < 18 years old. Focus groups were transcribed verbatim and analyzed using content and deductive thematic analysis. The capability opportunity motivation behaviour model was used as our conceptual framework.

**Results:**

Four virtual and three in-person focus groups were conducted with 47 participants. Overall, parents were motivated and willing to vaccinate their children but experienced several barriers related to their capability and opportunity to access RCVs. Five main themes emerged: 1) lack of reputable information about RCVs, 2) language barriers when looking for information and asking questions about RCVs, 3) lack of access to a primary care provider (PCP), 4) lack of affordable and convenient transportation options, and 5) due to the COVID-19 pandemic, lack of available vaccine appointments. Several minor themes were also identified and included barriers such as lack of 1) childcare, vaccine record sharing, PCP follow-up.

**Conclusions:**

Our findings highlight that several barriers faced by newcomer families ultimately stem from issues related to accessing information about RCVs and the challenges families face once at vaccination clinics, highlighting opportunities for health systems to better support newcomers in accessing RCVs.

**Supplementary Information:**

The online version contains supplementary material available at 10.1186/s12889-023-16258-7.

## Introduction

Vaccinations have held a critical place in human health and wellbeing since they were first developed. The Global Alliance for Vaccines and Immunization estimates around 1.5 million children die per year from vaccine preventable diseases [1]. Thus, it is imperative that children are up to date on their routine childhood vaccinations (RCVs). RCVs are immunizations children receive in their first 18 years of life to help protect them against various illnesses and contribute to overall child and community health [2]. In Canada, public perception of RCVs began to decline even before the COVID-19 pandemic, with a drop of 8.2% over the last 3 years [3]. It is well known that amongst historically underserved populations, such as newcomers (e.g., immigrants, refugees, temporary foreign workers), RCV rates tend to be much lower than the general population [4].

Newcomers make up 23% of the Canadian population, with Alberta being home to the third highest newcomer population where one in every third person belongs to a newcomer community [5, 6]. Newcomers face several barriers when accessing public health services in Canada such as issues accessing a primary care provider (PCP), coordinating childcare and transportation, and understanding which RCVs are offered in Canada and when, which have been shown to contribute to low vaccination rates amongst newcomer families [7–9]. It is important to note that, unlike other provinces in Canada, PCPs in Alberta do not routinely administer RCVs, but instead they are administered by public health nurses at public health clinics or school-based programs. As Immigration, Refugees and Citizenship Canada (IRCC) plans to increase immigration to ~ 500,000 people per year from 2023–2025 [10], it is important that the barriers faced by newcomer families are clearly understood so our health systems can learn how to better support these families as they transition to life in Canada. In addition, the province of Alberta has introduced an Express Entry Stream [11] which allows newcomers to enter Alberta more easily based on merits such as ties to Alberta (e.g., letter of employment) or proof of a post-secondary education degree, suggesting that Alberta could see an increase in the number of newcomers entering the province compared to other jurisdictions.

To identify ways to better support newcomers when accessing RCVs in Canada, it is important to first understand their current barriers and attitudes towards accessing RCVs. The Capability, Opportunity, Motivation behaviour (COM-B) model is used to frame action-oriented conversations to identify opportunities for improvement by identifying how an individual’s capability (knowledge base), opportunity (access or their lack of), and motivation (personal beliefs and values) affects their ability to change their behaviour [12]. Therefore, behaviour change models such as COM-B can be used to inform the development of community based-interventions to promote and support positive behaviour change, such as identifying ways to increase RCV uptake amongst newcomer families.

Therefore, the primary objectives of the study were: 1) considering the COVID-19 pandemic, understand newcomer parents’ attitudes towards RCVs, and 2) through the lens of the COM-B model, identify barriers newcomer parents face when accessing RCVs in Alberta, Canada.

## Methods

### Study design

We conducted focus groups between July 6th—August 31st, 2022 with 47 individuals in Calgary, Edmonton, and High River, Alberta, Canada. Focus groups were used to foster valuable discussions to identify key barriers and attitudes to accessing RCVs. We anticipated that this sample size would allow for adequate information power which considers that the more specific the study aim is, the more information the sample holds related to the topic, and therefore, a lower number of participants are required [13].

### Participant recruitment

Participants were recruited through partner agencies from the Calgary Catholic Immigration Society (CCIS) [14], a non-profit organization providing both settlement and integration services to newcomers in Southern Alberta; and the Multicultural Health Brokers Co-operative (MCHB) [15] in Edmonton, Alberta, a community led organization helping to bridge the gap between newcomers and their integration into Canadian society. Participants were recruited from both rural and urban regions in Alberta to ensure representation from newcomers living in rural areas, a demographic which has typically been underrepresented in research [16]. Participants were asked to participate in a focus group (online or virtual) to understand their barriers and attitudes towards RCVs, and to identify barriers and attitudes to accessing RCVs in Canada. Eligibility was defined as newcomers (including refugees, immigrants, and migrant workers) who migrated to Canada within the last five years [17], and had at least one child 18 years old and/or younger.

### Focus group guide development

The focus group guide was co-developed with partner agencies to ensure the guide was culturally appropriate and relevant. The COM-B model informed the focus group guide, and the research team conducted a literature review to identify known barriers faced by newcomers when accessing vaccinations. Recognizing that vaccine schedules and names may be different country-to-country, at the start of the focus group we listed out which RCVs are given to children in Canada and at what ages (Additional file 1: Appendix A). The focus group guide was written in English and translated into Tagalog, Arabic, Juba Arabic, Bangla, and Somali. Partner agencies helped the research team identify some of the most spoken languages amongst newcomers in Alberta. Upon translation, the guide was validated for accuracy by members from the partner agencies.

### Focus group guide moderation

Focus group discussions were led by members of the research team, including researchers from partner agencies who moderated focus groups in other first languages. Virtual focus groups were conducted online using Zoom (Zoom Video Communications, Inc., San Jose, CA) and in-person focus groups were conducted at newcomer serving organizations in Calgary, Edmonton, and rural Alberta, Canada. Focus groups were 1.5 h in length and were moderated by one researcher, while one to three facilitators observed, took notes, and provided support. Following the focus group, the moderator, and facilitator(s) debriefed and shared field notes.

### Qualitative analysis

In-person focus groups were audio recorded and virtual focus groups were audio and video recorded. The recordings were transcribed verbatim and translated (when appropriate), to support rigorous data analysis. Six qualitative researchers performed content analysis in the six first languages to identify themes. Analyzing the transcripts in first languages allowed researchers to ensure any significant emotions, sentiments, and ideas were captured appropriately. The themes and all participant quotes were then translated into English, where two qualitative researchers conducted in-depth deductive thematic analysis using the COM-B Model to identify common perceptions and opinions. Major themes were classified as themes and barriers that appeared the most often in discussion and through the analysis, whereas minor themes appeared less often and were expressed by only a few participants. A qualitative data analysis software, NVivo Qualitative Data Analysis Software (QSR International, Version 12) was used to support data organization and analysis. Regular communication between the data analysts ensured that ongoing changes to the analysis were discussed and agreed upon. The coding and thematic analysis was also supported by reviewing field notes recorded during each focus group, and checking the emergent findings with the focus groups facilitators to ensure no key themes were missed.

## Results

**Fig. 1:**
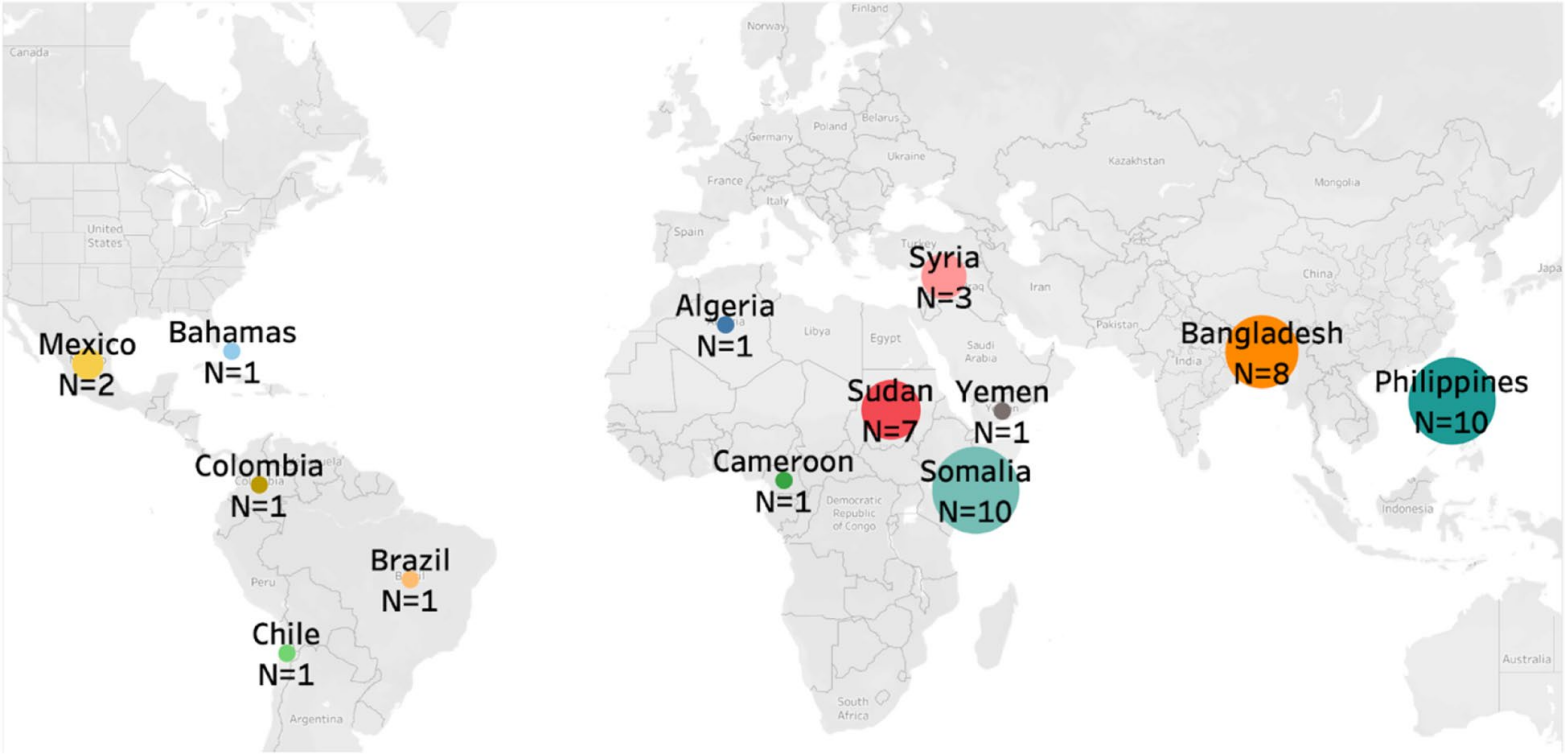
Focus group participant countries of origin including global representation from 13 countries

Seven 1.5 h-long virtual (*n* = 4) and in-person (*n* = 3) focus groups were conducted with 47 participants total with representation from 13 countries of origin (countries participants migrated to Canada from) (Table 1, Fig. 1). Overall, the participants included 40 females and 7 males who had been in Canada for 1 to 5 years, were between the ages of 22 to 53, had children aged > 1 year to 25 (with at least one child 18 or younger), and had 1 to 6 children/family.

**Table 1:** Focus group locations in Alberta, Canada including the languages the focus groups were conducted in (not all individuals participated in their first language)

Focus Group Number	Region	Language of Focus Group
1	Rural Alberta (*n* = 7)	English
2	Urban Calgary (*n* = 6)	English
3	Urban Calgary (*n* = 8)	Tagalog
4	Urban Calgary (*n* = 3)	Arabic
5	Urban Edmonton (*n* = 7)	Juba Arabic
6	Urban Edmonton (*n* = 6)	Bangla
7	Urban Edmonton (*n* = 10)	Somali

### Attitudes towards routine childhood vaccinations, including COVID-19 vaccines

Overall, most participants had very few reservations about vaccines, expressing that their decision to vaccinate their children resulted from trusting medical professionals and the science behind RCVs, and believing that vaccines play an important role in keeping their children healthy and safe.

*“I think that it's, uh, important to take vaccines because […] it's a way to stimulate their immune system and it's a science, so behind, behind that, so I believe in science” (Participant 8, Rural Alberta English).*

*“l believe that the vaccines are helpful and can protect my child from being severely sick […] with the vaccines, it can help my children fight the sicknesses […] the power of vaccines cannot be ignored” (Participant 37, Urban Edmonton Juba Arabic).*

In addition, participants expressed that their decision to vaccinate their children since arriving in Canada was largely due to their trust in and support for the Canadian public healthcare system, including the belief that advancements in immunizations are being made in Canada compared to other countries.

*“When we say we look here in Canada, there's people living to 80 or 90 years old, you know why [because] there [are] immunization there.” (Participant 15, Urban Calgary English).*

However, some participants had concerns with COVID-19 immunizations for children. This ultimately came down to three main reasons: 1) unsure of the long-term side-effects of the COVID-19 vaccines offered to children, 2) novelty of the virus, and 3) overall lack of trust in the COVID-19 vaccines. As a result, a few participants indicated that they would wait until more information became available about the long-term side-effects before deciding whether to vaccinate their children or not.

*“For me, now that they are saying that it’s available for four year-old and below, I am a bit hesitant about the COVID vaccine because we don’t know much about COVID, it’s so new” (Participant 16, Urban Calgary Tagalog).*

*“The COVID vaccine is a controversial issue right now.” (Participant 14, Urban Calgary English).*

Although some parents had concerns about COVID-19 vaccines, it is important to highlight that these concerns did not seem to affect their attitudes or behaviours towards RCVs during the pandemic.

*“[…] in general, for the general vaccines, I don't have any concern. But, uh, regarding the COVID vaccine, I don't know if I will, um, do it for my child. I think I'm gonna wait some years till it's [proven] that there [are] no tiny, um, side effects.” (Participant 11, Urban Calgary English).*

### Key barriers to accessing routine childhood immunizations in Alberta, Canada

Overall, five major themes emerged when identifying key barriers faced by newcomers when accessing RCVs in Alberta, Canada, 1) lack of reputable information about RCVs, 2) language barriers when looking for information and asking questions about RCVs, 3) lack of access to a PCP, 4) lack of affordable and convenient transportation options, and 5) lack of available vaccine appointments (Table 2).

**Table 2:** Five key barriers experienced by newcomer parents when accessing RCVs in Canada

Key Barrier	Sub-Theme	Key Quotes
**1. Lack of access to reputable information**	**General feelings of not knowing where to begin when searching for information about RCVs**	*“Information is not readily available about which clinics to go for which vaccines and lack of fluency between information available online, the clinics, and peoples interested.” (Participant 46, Urban Edmonton Bangla)* *“In our case, we were the ones who initiated that, “doc, these are the vaccines [that our child has], what do we have to do next? […] the initiative was coming from us.” (Participant 18, Urban Calgary Tagalog)*
	**Parents are seeking easy to access vaccine schedule information**	***“****I receive a, um, a, like a card, blank, and then the doctor will record the immunizations, uh, from that, in Ontario when we were there. So, but here in Edmonton, I didn't get that, so that's the challenge. Not knowing what, what is next.”(Participant 53, Urban Edmonton Somali)*
	**For non-permanent residents, it is challenging to access health cards which are needed at RCV appointments (minor theme)**	*“Health Cards are only valid for 1 year so this creates a rush to get vaccines and man worries about obtaining other health-care services”—(Participant 46, Urban Edmonton Bangla)*
	**Parents generally would like to learn more about the Canadian healthcare system (minor theme)**	*“Even though I had knowledge of vaccines back home, just, you know, the change of the, the place and the name of the vaccines in English and many of the aliases in English, all that can be, uh, so I guess it's some kind of lack of knowledge, of how the Canadian system works in terms of vaccines, that can be also another barrier.” (Participant 48, Urban Edmonton Bangla)*
	**Parents would like to be provided with more information about vaccine side effects (minor theme)**	*“So now people are kind of suspicious to avoid, even they might take the children to b- back home, doing the, some of the, uh, vaccine, so w- we need education. I mean, the professional edu- educators, specially when it comes to vaccine for the children, because w- I mean, the communities afraid of the, having the side effects.” (Participant 38, Urban Edmonton Juba Arabic)* “The side effect of the vaccine, cause my, my young youngest, um, every time he has a vaccine, he always, um, got fever and um, and, and I'm having a hard time giving him a medicine for that. That's why I'm when his vaccine schedule comes again. I had, I always have a second thought if I, I will bring him to the public health.” (Participant 7, Rural English)
2. Challenges communicating in English	**Parents struggle with not being able to speak/ understand English as well as not always having access to a translator**	*“They [healthcare providers] used to talk to us, and we didn't understand what they were saying” (Participant 35, Urban Calgary Arabic)* *Some health care providers are hesitant to navigate tricky language based situations with non-English speaking patient. For example, service providers aren't willing to understand different accents…He didn't even want to understand what I was talking about” (Participant 45, Urban Edmonton Bangla)*
3. Lack of access to a PCP who is accepting patients	**Identifying and accessing a family doctor who is taking patients is frustrating for parents who have questions about RCVs**	*“But when my, uh, youngest daughter was born I didn't get much guidance from the hospital [with vaccines]. Um, and then it took me a while to find a family doctor (laughs) when we, when we were in Toronto” (Participant 8, Rural Alberta English)*
4. Lack of affordable and convenient transportation options	**For parents who do not have access to a personal vehicle, it can be very challenging to rely on public transit**	*“The major problem is that going with young children in a public transport is suicidal […] It is hard to control the kids. At the end of the day transport becomes a problem […]”(Participant 38, Urban Edmonton Juba Arabic)* *“The second [barrier] is transportation during the winter months and sometimes having to wait for 45 min [for public transit] while it is 25 degrees below zero” (Participant 36, Calgary Arabic)*
	**Some families rely on working husband to drive family to vaccine appointments (minor theme)**	*“I am learnt in Arabic and had so many challenges with the language and transport since my husband used the vehicle for work. He did not tale time off and l was really stressed out. I wish the nurses could offer transport. I did not ask, and l am not aware of any transport to help me out” (Participant 36, Edmonton Juba Arabic)*
5. Lack of available vaccine appointments during the COVID-19 pandemic	**Due to COVID-19, it has been harder to find available vaccine appointment times**	*“The only challenge I had here is when I had to book the, uh, two years vaccine for my babies […] they didn't give me the same time because they are always fully booked and [couldn’t take them both] at the same time.” (Participant 26, Rural Alberta English)*
	**Those living in rural communities find it easier to make last minute vaccine appointments (minor theme)**	*“COVID was very easy to take because, um, it's everywhere in the pharmacies, in the clinics it's everywhere. But for the other ones, you have to book an appointment. But because High River is very small, so you can go easily, even if you wanna walk or okay. Ask for a ride.” (Participant 9, Rural English)*

### Access to information

Overall, most participants were unsure of “where to start” to look for and access RCV information in Alberta, Canada, as they find the process difficult to navigate. As a result, participants would like to be better informed and educated on the Canadian public health system when first arriving in Canada families may follow through with RCVs more confidently.

*“It is hard for a newcomer without any connection with those from their community [to] obtain vital information and support for important [health] decisions” (Participant 37, Urban Edmonton Juda Arabic).*

Participants found it challenging to access information about Canadian vaccination schedules (e.g., ages children receive specific vaccines) as the schedules may not align with when their children would have received their RCVs in their country of origin. Therefore, participants wished that reputable websites such as the IRCC, provided better information on RCVs.

*“I still think that doctor, uh, the doctor could have given me a list of the vaccines and where I would get them […] yeah, that, that, that's the missing part […]”* (Participant 11, Urban Calgary English)

### Language barriers

Participants identified their inability to speak/understand English or communicate in their first language(s) when trying to access information and talk to healthcare providers about RCVs as a major barrier. Participants found it frustrating and upsetting when translators were not available to support them in their first language at public health clinics and left to communicate their questions and concerns (e.g., side effects to RCVs) with a provider who does not understand them.

*“… the interaction were not great due to language barrier. I can speak English but when with the nurse l need a slow talk to understand the nurse.” (Participant 40, Urban Edmonton Juba Arabic).*

### Access to a PCP

For families new to Canada, it was very difficult to find who was accepting patients. Not having access to a PCP was one of the main access barriers inhibiting participants from asking questions about vaccines as they struggled to access translatable material elsewhere. For those fortunate enough to find a PCP, they believed that PCPs should be better equipped to answer questions about RCVs and support parents about the RCV schedule (e.g., reminding parents when their children are due for their next vaccine).

*“I didn't get enough information from my family physician office […] I think if, the adequate information should be in the family physician's office so that we can access that” (Participant 44, Urban Edmonton Bangla).*

### Transportation

Another barrier participants experienced was the lack of accessible transportation options to RCV appointments for those who did not have access to a personal vehicle, or public health clinics located in hard-to-reach locations.

*“I [speak]Arabic and had so many challenges with the language and transport since my husband used the vehicle for work. He did not take time off and l was really stressed out. I wish the nurses could offer transport. I did not ask, and l am not aware of any transport to help me out” (Participant 36, Urban Edmonton Juba Arabic).*

### Availability of vaccine appointments

Lack of available vaccination appointments and limited hours of operation at public health clinics made it difficult for participants to get their child vaccinated as appointments were often weeks/months in advance. A few participants expressed that they believed this was a result of the COVID-19 pandemic and that prior to the pandemic they could make an appointment much faster.

*“But after COVID, that’s when it became harder [to get regular vaccinations]. For my second baby, it was hard to book, waiting time was also worse. And only one parent could go with the child. If my child has a runny nose, they won’t let you in.” (Participant 17, Urban Calgary Tagalog).*

### Other barriers experienced by newcomer parents

Several minor themes also emerged when exploring barriers experienced by participants when accessing RCVs in Alberta, Canada. Other barriers experienced by some of the participants included, not being able to take time off work, lack of PCP follow-up, lack of childcare during appointments, unpleasant interactions with PCPs, religious beliefs, a partner who does not believe in vaccines, and record sharing between countries. These minor themes are explored further in Table 3.

**Table 3:** Other barriers experienced by newcomer parents when accessing RCVs in Alberta, Canada

Barrier	Minor Theme	Key Quotes
**Inability to take time off work to attend RCV appointments**	**The ability to take time off work to attend a child’s vaccination appointment is not an option as most clinics are during working hours**	*“My wife made the appointment, and my l was able to take time off from work as sick day to look after the health of my children” (Participant 38, Urban Edmonton Juba Arabic)* *“And you're able to take that time off work?”… “No, it depends. Sometimes I can sometimes I can't.” (Participant 9, Rural English)* *“Time slots for vaccines overlap with working hours and require coordination with my child's schedule” (“12–8” and “9–5″ are hard times to accommodate” (Participant 47, Urban Edmonton Bangla)*
**Lack of PCP or public health follow-up**	**Parents rely on healthcare providers to follow-up and remind them of when their child is due for their next dose and/ or vaccine**	*“In our case, we were the ones who initiated that, “doc, these are the vaccines [that our child has], what do we have to do next?” The initiative was coming from us.” (Participant 18, Urban Calgary Tagalog)* *“There’s also an unfriendly nurse there. For example, you had your vaccine now and you ask her/him if s/he could remind you about your next appointment, I was told “That’s your responsibility.” (laughs) I was just only asking…of course, if it’s like that, you won’t ask anymore.” (Participant 17, Urban Calgary Tagalog)*
**Lack of childcare during appointments**	**It can be overwhelming for parents if they are not able to find childcare and must resort to bringing their other children to vaccination appointments**	*“I had to struggle with my second baby's vaccination. I used to take my elder daughter with us during my little one's vaccination as there was no one at home to look after her. My elder daughter was not happy at all as we had to wait for a little while and there was no play zone in the vaccine centre for little kids.” (Participant 46, Urban Edmonton Bangla)* *“Wait times at clinics and lack of entertainment and support for kids while at the clinic made the experience challenging” (Participant 48, Urban Edmonton Bangla)* ***During the COVID-19 pandemic families were not allowed to bring more than one child to appointments*** *“Family members and siblings who take care of supervision were not allowed inside vaccine clinics” (Participant 48, Urban Edmonton Bangla)*
**Unpleasant interactions with PCPs**	**Unpleasant interaction with health providers may discourage parents from seeking information about vaccines**	*“Our family doctor…seems to be always in a rush (laughter). S/he talks very fast and doesn’t provide us with any information [about vaccines]. Only when there’s requisition for blood works, s/he will call you. Once you’re there, s/he will tell you (inaudible)..that’s it… (translated)” (Participant 17, Calgary Tagalog)*
**Recording sharing between different jurisdictions and countries**	**The lack of health data record sharing between different health systems (e.g., country to country), and was frustrating for participants when they were trying to track and access their child's vaccination records as well as show proof of vaccination (e.g., at schools)**	*“I came here, my kids are four months old. And I don't have the record of what they get back home. When I come here…I have difficulties explaining to them [public health] because I don't have any [vaccine] records. And they tell me that they are gonna start from the beginning. And I don't know if they duplicate some vaccines. I don't know it can affect them or not” (Participant 53, Urban Edmonton Somali)* *“ …the issue of, uh, documentation. My youngest son was vaccinated in Uganda with all [vaccines]…but when he came here there was no documentation… (Participant 46, Urban Edmonton Bangla)”* *“But in school he was threatened with exclusion that, I mean, if there was any outbreak he would be excluded. And so we had to go through what we had already, uh, obtained, I mean, have him re-vaccinated.” (Participant 43, Urban Edmonton Bangla)*
**Partner who does not believe in vaccines**	**Partners who are vaccine hesitant, or do not believe in vaccines often result in the other partner not pursuing RCV for their children**	*“Based on my experience, because my husband is older…we were ok during our time even if we didn’t have vaccines…It’s like there’s lack of knowledge about the vaccine. For me as a midwife, I know this is very important. For him, he would think that in the past, you survived without vaccine…he’s afraid that his child would get sick because vaccines have side effects. If our child would have fever, he’d be scared. But if you read the instructions, you would know that side-effects would be within two weeks. But he would think, he would panic. I would tell him, “It’s ok, that’s side effects.” So yes, lack of knowledge for him. For me, [vaccine] it’s very important” (Participant 17, Calgary Tagalog)*
**Religious beliefs that may prevent parents from vaccinating**	**Parents are unsure if their religion will allow RCV (e.g., unsure if the vaccine ingredients contain pork)**	*“But some challenges also could be, uh, believing that what is in the vaccine, is this gonna be something that my religion allows? Is there a pork in it, to Islamic culture? They say, well it has. Is there, you know, like, something that is against my belief” (Participant 34, Urban Calgary Arabic)*

## Discussion

**Fig. 2:**
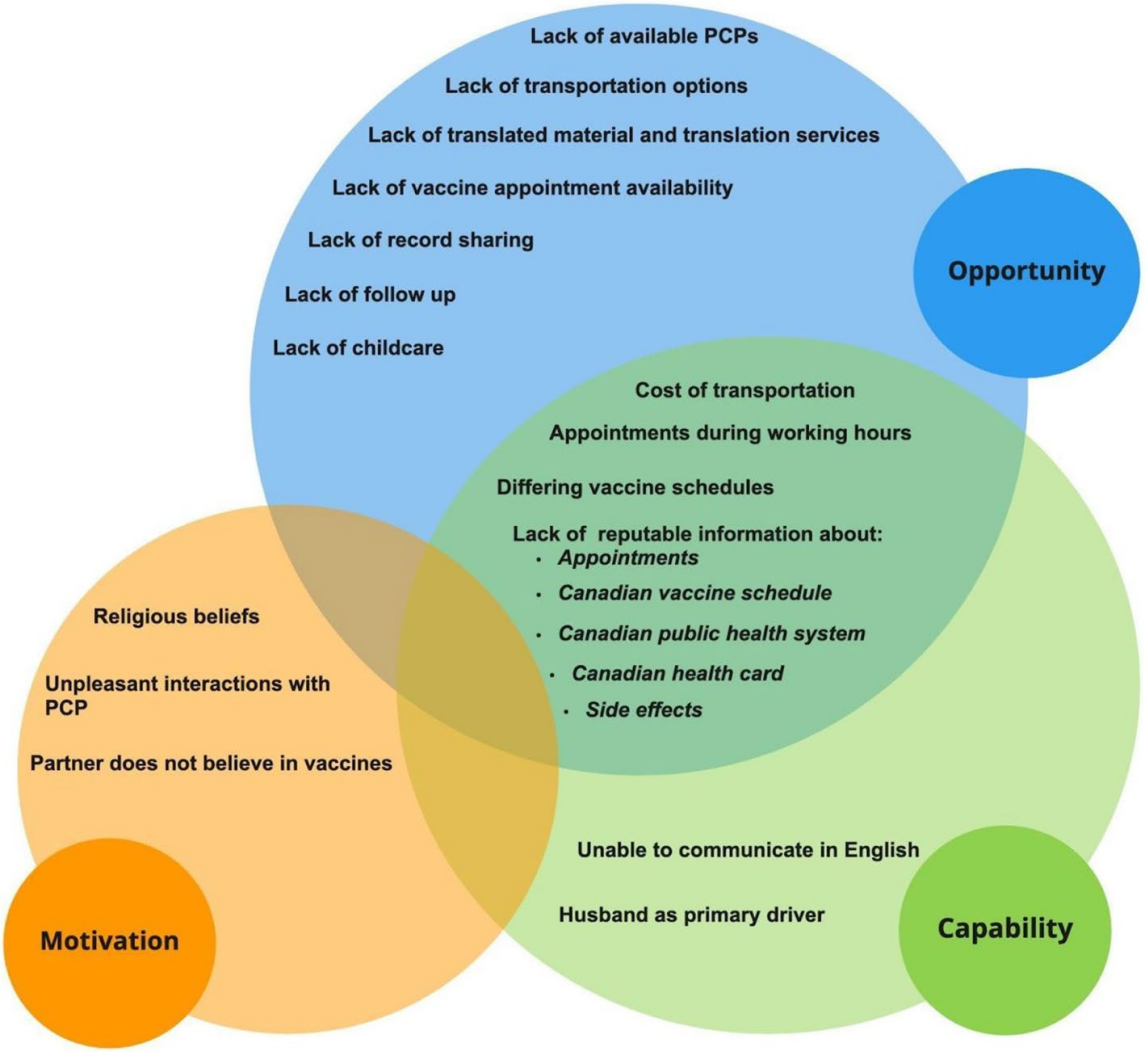
Participant barriers to receiving RCVs through the lens of the COM-B model for behaviour change

In light of the COVID-19 pandemic, newcomer parents were supportive of RCVs and ultimately, their decision to vaccinate their children was based on how long RCVs have been around for, including the belief that vaccines play an important role in keeping their children healthy and safe. Although some parents had concerns about COVID-19 vaccines this did not seem to impact their attitude towards RCVs during the pandemic. By mapping the barriers identified in this study to the COM-B Model (Fig. 2), we determined that most of the barriers experienced by newcomer parents related to the lack of opportunity (e.g., external factors that make a behaviour possible) and their individual capability (e.g., an individual’s psychological and physical ability to participate in an activity) to access RCVs in Canada, and had less to do with their motivation (conscious and unconscious cognitive processes that direct and inspire behavior) or willingness to vaccinate [12]. We identified barriers related to one’s opportunity and capability such as lack of access to information about RCVs (e.g., information about booking appointments, vaccine schedules (as they may vary from other countries), side effects), language barriers (e.g., accessing translated material, translators, and inability to communicate in English), lack of record sharing between health systems (e.g., country to country), and lack of access to a PCP who participants believed could answer their questions, get to know the needs of the family and child, and ultimately, support them through the RCV process. Several of these barriers can be attributed to the closures of public health clinics and school-based programs during the COVID-19 pandemic, which made it harder to access RCVs. Especially for those who had migrated to Canada just before or during the pandemic and were not familiar with the vaccination schedule in Alberta.

In agreement with our findings, a recent systematic review [7], identified similar barriers related to one’s opportunity and capability such as lack of record keeping, cost of transportation to get to vaccine appointments, challenges finding childcare for siblings, availability of vaccine appointments, wait times once at the clinic, with the most prominent access barrier being lack of information about RCVs. It is evident that many of the barriers experienced by newcomers when accessing RCVs stem from not being able to access reputable sources of information. Considering the COVID-19 pandemic, this may be a result of the “infodemic” which has had a significant impact on vaccine information [18] and shines a spotlight on the need to help newcomers cut through the noise to identify reputable sources to stay up to date and informed.

Moreover, very few parents lacked the motivation to seek out information about RCVs or to access public health clinics where vaccines are administered. However, minor themes identified related to one’s motivation to vaccinate included unpleasant interactions with PCPs, religious beliefs, partners who do not believe in vaccines (Fig. 2). In previous studies, an individual’s motivation, or their lack of, seemed to have played a much larger role in their decision to vaccinate compared to our findings, for reasons such as concern about vaccine safety, and lack of trust in the government, vaccines, and PCPs [19]. Few individuals who participated in our study appeared vaccine hesitant and expressed concerns about vaccines, but this was mainly in relation to concerns towards COVID-19 vaccines and not RCVs.

By addressing some of these external factors, our findings suggest that there is an opportunity to increase RCV uptake amongst newcomer families who are already motivated to vaccinate their children but lack the capability or opportunity to do so. Previous work [20] identified that Alberta’s communication strategies used to inform newcomers about RCVs are ineffective at reaching newcomers; however, when individuals such as health brokers guided families in accessing information and provided them with the supports to do so, they were likely to receive RCVs [20]. It is also known that newcomers to Canada are more likely to participate in vaccinations compared to non-newcomer populations [21], with evidence showing that simple interventions such as emphasizing PCPs as vaccine educators [22], appointment reminders in first languages [23], providing transportation to vaccine clinics or helping with the cost [24, 25], food vouchers for attending RCV appointments [26], can be highly effective at increasing RCV uptake amongst newcomer families. Therefore, in the context of Alberta, where RCVs are solely administered by public health nurses in public health settings, it is important to work with all health systems (e.g., public health, PCPs) involved in administering and promoting RCVs to ensure that the barriers experienced by newcomers are addressed in a meaningful way. This includes working with partner agencies (e.g., settlement organizations) who are trusted voices amongst newcomers as they are often the first contact for newly arrived newcomer families in Canada [20].

The main strength of this study was the ability to conduct focus groups in multiple first languages to promote inclusion of ethnocultural minorities in public health research (e.g., participants and researchers). Previous studies with newcomers, conducted in other high-income countries that receive immigrants, have mainly been completed or translated into English, making our current study distinctive. There are several study limitations. Commonly seen in focus groups, recruitment bias and social desirability bias must be taken into consideration. Participants recruited from CCIS and MCHB were already engaged in these organizations and motivated to participate and express their thoughts about vaccines, an increasingly prevalent topic. Similarly, with social desirability bias, participants may have said what they felt was necessary to stay aligned with the groups and moderator’s morals or values. Most participants were seemingly vaccine confident, with little hesitation about the effectiveness or importance of RCVs, possibly indicating that social desirability bias may have played a role in these pro-vaccine views. Despite the overall goal of discussing RCVs, the topic of COVID-19 vaccine was more prominent in some focus groups than others. We considered conducting focus groups in multiple first languages a strength; however, questions and answers may not have been translated or analyzed within the same context as English. Lastly, given that RCVs are primarily administered in public health clinics, the results from our study can not be generalized outside of Alberta where RCVs are offered in multiple settings by different providers.

## Conclusion

Using the COM-B model as our framework, we identified key barriers experienced by newcomer parents when accessing RCVs in Alberta, Canada. Ultimately, our results suggest that amongst newcomers who are already motivated to vaccinate their children, there are opportunities to reduce access barriers related to capability and their opportunity to access RCVs. In particular, most of the barriers identified in this study stem from issues related to accessing reputable sources of information about RCVs and the challenges families face once at public health clinics. Therefore, some barriers may be easier to address than others but ultimately, it is important to recognize that all groups within the health system need to be engaged to move forward in an action-oriented way. This includes working alongside community organizations who have pre-existing relationships with their community members and are better positioned to purposefully reach newcomer families. As Canada and other high-income countries continue to welcome newcomers, it is important that we continue to identify ways to improve their transition to new health systems and ultimately, help support a healthier community for all.

## Supplementary Information

**Additional file 1: Appendix A. **Focus Group Guide.

## Data Availability

The datasets used and/or analysed during the current study available from the corresponding author on reasonable request.

## Abbreviations

RCVs: Routine-childhood vaccinations
PCP: Primary care provider
IRCC: Immigration, Refugees and Citizenship Canada
COM-B: Capability, Opportunity, Motivation behaviour
CCIS: Calgary Catholic Immigration Society
MCHB: Multicultural Health Brokers Co-operative
