# Supplementary material for: Barriers experienced by families new to Alberta, Canada when accessing routine-childhood vaccinations
Source: BMC Public Health. 2023 Jul 12;23:1333. doi: 10.1186/s12889-023-16258-7 (PMC10337205; doi:10.1186/s12889-023-16258-7)
Supplement: Supplementary file 1 — Additional file 1: Appendix A. Focus Group Guide. [file 12889_2023_16258_MOESM1_ESM.docx]

**Focus Group Script and Questions**

**Initial dialogue**:

*Thanks everyone for taking the time to participate in this focus group today.* *My name is __________and I’m going to lead our discussion today by asking you questions, as well as encouraging and moderating our discussion.*

*This will be a 90 minute focus group about routine childhood vaccinations - these are particular vaccinations that children receive on a specific schedule to help keep them safe and healthy. Today we will talk about challenges you faced in getting your children's vaccinations, as well as things that made or could make the process easier. This is a confidential discussion; we ask that you respect the privacy of everyone involved by not discussing what was shared, beyond this focus group. What is talked about today will be used to help develop evidence-based, tailored interventions to increase vaccine uptake among children new to Canada. All answers will be summarized into themes to ensure that no individual can be identified. This discussion will be audio recorded to allow us to go back and identify themes and analyze answers, but only those involved with the analysis will have access to these recordings.*

*There are no right or wrong answers, just different views, practices, and reasons for why people choose to do what they do. We will ensure everyone has some time to respond to each question, before encouraging some general discussion. You are encouraged to be honest and share your true thoughts and feelings, even if you disagree with others in the group; however, please be respectful and listen without interrupting when others are speaking.*

*Please share YOUR thoughts and ideas, not those of friends or family. If at any time you feel uncomfortable or wish to no longer participate in the discussion, you are free to do so.*

*Before we get started, does anyone have any questions?*

**Icebreaker**

Please introduce yourself using only your first name, initials, or an alias; then answer this question.

- What is your favourite summer activity?

**Script - Round table discussion 60 mins**

Internal Notes for Moderator:

- 2 months: Diphtheria, tetanus, acellular pertussis, polio, haemophilus influenzae type b, hepatitis b, Pneumococcal conjugate, Rotavirus
- 4 months: Diphtheria, tetanus, acellular pertussis, polio, haemophilus influenzae type b, hepatitis b, Pneumococcal conjugate, Meningococcal conjugate, Rotavirus
- 6 months: Diphtheria, tetanus, acellular pertussis, polio, haemophilus influenzae type b, hepatitis b, Rotavirus, influenza
- 12 months: Measles, mumps, rubella, and varicella, Pneumococcal conjugate, Meningococcal conjugate
- 18 months: Diphtheria, tetanus, acellular pertussis, polio, haemophilus influenzae type b, Measles, mumps, rubella, and varicella
- 4 years: Diphtheria, tetanus, acellular pertussis, polio, Measles, mumps, rubella, and varicella
- Grade 6: Hep B, HPV
- Grade 9: Diphtheria, tetanus, acellular pertussis, Meningococcal Conjugate

**Do you *generally* know what vaccines are recommended for your children?**

**Do you believe these vaccines are important? If so, why?**

**Do you have concerns with vaccine safety or how effective it is? If so, why?**

**What sort of things influenced you to get the vaccines that are recommended to your children?**

*Prompts*

- Conversation with a family physician

*These next set of questions focuses on barriers to vaccination.*

**Have you been able to receive all of these vaccines for your children in your country of origin or in Canada?**

*Prompts*

- Where have your children received them?
  - GP?
  - Public health clinics?
  - School-based vaccination programs?

**What are some of the challenges you have encountered with accessing vaccines that are recommended for your children? Or what things have made it easier for you?**

*Prompts*

- What are your experiences with…
  - **Knowing when to book your vaccine appointments?**
    - **Things to consider:**
      - How do you find out when you need appointments (e.g., PH, friends/family, family doctor, etc.)?
      - Where do you generally get vaccine information from (e.g., PH, friends/family, family doctor, etc.)?
  - **Arranging the appointments?**
    - **Things to consider:**
      - Booking with public health, are appt times okay (e.g., evening/weekend appointments)
      - Appointment logistics (e.g., child care, transportation, enough time to go to appt based on other obligations like work, etc.)
  - **Once at the clinic?**
    - **Things to consider:**
      - Wait times, interaction with admin/secretary etc.
  - **The healthcare provider?**
    - **Things to consider:**
      - Poor communication / language skills, lack of knowledge, extent to which questions are answered, etc.
  - **Knowing what to expect after the appointment?**
    - **Things to consider:**
      - Being aware of and knowing how to respond to side effects, follow-up questions, etc.

**What are some ways that the vaccination process could be improved for you?**

*Prompts*

- Vaccination centre location
- Access to transportation
- Transportation costs
- Total time spent receiving a vaccine
- Waiting times at a vaccination centre
- Ease of booking an appointment
- Interactions with providers

**Over the past two years there has been a large spotlight on vaccines, because of the COVID-19 pandemic. Do you have any new thoughts about vaccines, especially non-COVID-19 vaccines?**

*Prompts*

- How have your perspectives changed?
- Have your perspectives remained the same?

*The next few questions are about school-based vaccination programs.*

**[TIME DEPENDENT] Additional questions about school-based vaccination programs**

**What are your thoughts about school-based vaccination programs?**

*Prompts*

- For example, the HPV vaccine (Human papillomavirus vaccines are vaccines that prevent cervical cancer) is available for school-aged children to receive in Junior High at school - do you think it is a good idea to give vaccines in schools?
- What are your thoughts on offering COVID-19 vaccinations in schools?

**How do you think schools could improve vaccine communication?**

*Prompts*

- What resources would you like to see?
  - Around the school, in school newsletters
- Would you attend an information session/open Q&A to get informed and have your questions answered?

**Individual Exercise - 30 mins (including discussion)**

*Now, we are going to do a short exercise. We will be handing out a piece of paper (if online, it will be emailed to you). We have made a list of all the things that were talked about that make it challenging to get your children their vaccinations. We would like you to think about and write down the three MOST important challenges for you. {Give them time to complete this exercise}*

*If you are comfortable, please share why these were your most important challenges.*

**Closing Comments:**

*Does anyone have any final comments or thoughts they would like to share?*

*Thank you for taking part in this focus group, your discussion has been very helpful and appreciated. We are grateful to you for taking the time and sharing your experience with this group. If after we are done, there is something you still wish to share, or did not have the opportunity to discuss today, please pass it along to [INSERT RECRUITER].*
